# Fitness, Fatness and Active School Commuting among Liverpool Schoolchildren

**DOI:** 10.3390/ijerph14090995

**Published:** 2017-08-31

**Authors:** Robert J. Noonan, Lynne M. Boddy, Zoe R. Knowles, Stuart J. Fairclough

**Affiliations:** 1 Department of Sport and Physical Activity, Edge Hill University, Ormskirk L39 4QP, UK; Stuart.Fairclough@edgehill.ac.uk; 2 Physical Activity Exchange, Research Institute for Sport and Exercise Sciences, Liverpool John Moores University, Liverpool L3 2AB, UK; Lynne.M.Boddy@ljmu.ac.uk (L.M.B.); Zoe.R.Knowles@ljmu.ac.uk (Z.R.K.); 3 Department of Physical Education and Sport Sciences, University of Limerick, Limerick V94 T9PX, Ireland

**Keywords:** child, active commuting, physical activity, fitness, weight, obesity, neighborhood, deprivation, poverty, obesogenic

## Abstract

This study investigated differences in health outcomes between active and passive school commuters, and examined associations between parent perceptions of the neighborhood environment and active school commuting (ASC). One hundred-ninety-four children (107 girls), aged 9–10 years from ten primary schools in Liverpool, England, participated in this cross-sectional study. Measures of stature, body mass, waist circumference and cardiorespiratory fitness (CRF) were taken. School commute mode (active/passive) was self-reported and parents completed the neighborhood environment walkability scale for youth. Fifty-three percent of children commuted to school actively. Schoolchildren who lived in more deprived neighborhoods perceived by parents as being highly connected, unaesthetic and having mixed land-use were more likely to commute to school actively (*p* < 0.05). These children were at greatest risk of being obese and aerobically unfit (*p* < 0.01). Our results suggest that deprivation may explain the counterintuitive relationship between obesity, CRF and ASC in Liverpool schoolchildren. These findings encourage researchers and policy makers to be equally mindful of the social determinants of health when advocating behavioral and environmental health interventions. Further research exploring contextual factors to ASC, and examining the concurrent effect of ASC and diet on weight status by deprivation is needed.

## 1. Introduction

Childhood obesity and poor health are most prevalent in areas of high deprivation [1,2,3]. Physical activity (PA) improves child health, including weight status [4,5] and cardiorespiratory fitness (CRF) [6]. Active school commuting (ASC) is recognized as an important component of PA and is associated with higher daily PA [7,8]. In England, ASC prevalence among schoolchildren has progressively declined since 1995 [9], but remains consistently highest among schoolchildren from deprived backgrounds [10,11,12].

In recent years, there has been an increasing focus by the UK government to promote and increase ASC among schoolchildren with a view to curbing rising obesity levels [13]. However, evidence to support the positive contribution of ASC to children’s weight status is inconsistent [14,15]. For example, Voss and Sandercock [16] found no association between ASC and weight status whereas other studies have reported a weak inverse [17,18] and positive association [19]. The effect of ASC on other components of physical health such as CRF are also inconsistent [20,21]. Studies that have reported a positive association have been conducted outside of the UK in countries that experience greater cycling prevalence during ASC. Cycling is a stronger predictor of CRF in comparison to walking which is the most common form of ASC among UK children [16,22,23]. Therefore, further research is needed to explore the contribution of ASC to UK schoolchildren’s health.

ASC is influenced by multiple environmental factors. Household distance to school is considered the strongest influence with shorter distances associated with higher levels of ASC [24,25,26]. However, parents’ assessment of environmental attributes related to safety are also known to play an important role in determining whether children commute actively to school [27,28]. Neighborhoods perceived by parents as having well-connected streets, good land-use mix and residential density have been linked with higher ASC [29,30]. However, these reported associations are based on data from the USA [27,30] and Australia [31], which limits generalization to UK children. To promote and support ASC among UK schoolchildren, it is important to understand which environmental attributes support and restrict ASC. The Neighborhood Environment Walkability Scale-Youth (NEWS-Y), developed by Rosenberg et al. [32] provides an empirically derived measure of various built environmental attributes that may influence ASC. The NEWS-Y has been used to investigate associations between parental perceptions of the neighborhood environment and child PA [33,34] but not ASC. Therefore, the aims of this study were to (1) investigate differences in health outcomes between active and passive school commuters; and (2) examine associations between parent perceptions of the neighborhood environment and ASC.

## 2. Materials and Methods

### 2.1. Participants

Study participants were 9–10 years-old schoolchildren recruited from ten primary schools in Liverpool, England. Liverpool is ranked the most deprived English city [35] and obesity rates among children aged 10–11 years exceed the national average (23.0% vs. 18.9%; [36]). All eligible participants (*n* = 326) in participating schools received a participant recruitment pack containing parent and child information sheets, consent and assent forms, and a medical screening form. Written informed consent and assent were received from parents and their children, respectively, before children could participate in the study. Completed informed parental consent and child assent were obtained for 217 children (39.5% response rate). Liverpool John Moores University Ethics Committee approved the study (13/SPS/048) and data collection took place between January and April 2014.

### 2.2. Measures

#### 2.2.1. Anthropometrics

Stature and sitting stature were measured to the nearest 0.1 cm using a portable stadiometer (Leicester Height Measure, Seca, Birmingham, UK). Leg length was calculated by subtracting sitting stature from stature. Body mass was measured to the nearest 0.1 kg using calibrated scales (Seca, Birmingham, UK). Body mass index (BMI) was calculated from stature and body mass as a proxy measure of body composition (kg/m^2^) and BMI z-scores were assigned to each child [37]. Age and sex-specific BMI cut-points were used to classify children as normal weight or overweight/obese [38]. Waist circumference was measured at the midpoint between the bottom rib and the iliac crest to the nearest 0.1 cm using a non-elastic measuring tape (Seca, Birmingham, UK). Gender-specific regression equations were used to predict children’s age from peak height velocity (APHV; [39]). This calculation was used as a proxy measure of biological maturation.

#### 2.2.2. Cardiorespiratory Fitness

CRF was assessed using the Sports Coach UK 20 m multistage shuttle run test (20mSRT; [40]. Children completed 20 m shuttle runs keeping in time with an audible ‘bleep’ signal. The time between bleeps progressively decreases, increasing the intensity of the test. Children were encouraged to run to exhaustion, and the number of completed shuttles was recorded for each participant and retained for analysis. Age and sex specific cut-points were used to classify children as ‘fit’ or ‘unfit’ [41].

#### 2.2.3. School Commute Data

School commute mode was child-reported. Responses included (walk, cycle, scooter, bus, car, train, taxi, other). Responses were dichotomized into (0 reference category) active transport and (1) passive transport. Household distance to school was objectively measured using Google maps online route planner (https://www.google.co.uk/maps). The shortest route from school addresses to parent-reported home addresses was used [42].

#### 2.2.4. Neighborhood Environment

Parental perceptions of neighborhood attributes were assessed using the Neighborhood Environment Walkability Scale for Youth (NEWS-Y). The NEWS-Y is a 67-item scale, organized into nine subscales representing land-use mix diversity, neighborhood recreation facilities, residential density, land-use mix-access, street connectivity, walking/cycling facilities, neighborhood aesthetics, pedestrian and road traffic safety, and crime safety. The NEWS-Y has demonstrated acceptable to good test–retest reliability (ICC = 0.56–0.87; [32]) and has been used previously in child PA research [33,34]. Items are averaged and higher scores denote higher walkability. Higher neighborhood scores indicate a more walkable environment for all items except pedestrian and road traffic safety, and crime safety items, where higher scores indicate lower walkability [32]. An overall NEWS-Y score was calculated from the sum of *z*-scores for each of the nine subscales.

#### 2.2.5. Deprivation

Area level deprivation was calculated using the 2015 indices of multiple deprivation (IMD; [35]). The IMD is a UK government-produced measure comprising seven areas of deprivation (income, employment, health, education, housing, environment, and crime). Parent reported home postcodes were imported into the GeoConvert application [43] to generate deprivation scores. Higher deprivation was represented by lower deprivation scores. Sixty-eight percent of the study sample were above the IMD cut-off value (26.83) for the most nationally deprived tertile for England. We calculated a 50th centile IMD score of 35.63 for the sample, and created one IMD median-split categorical variable to provide two groups representative of children living in areas of high-deprivation (HD; median IMD score 49.76) and high-to-medium deprivation (MD; median IMD score 22.86; [34]).

### 2.3. Analysis

Participant characteristics were analyzed descriptively. Independent sample *t*-tests and χ^2^ compared descriptive data between genders. For study aim 1, multivariate analysis of covariance (MANCOVA) assessed differences in health outcomes by school commute mode (active vs passive) adjusted for gender, APHV, and school commute distance. χ^2^ with odds ratios (OR) as a measure of effect examined school commute mode group differences in weight status, aerobic fitness, deprivation and school commute distance. The same analyses were repeated to examine deprivation group differences in weight status, aerobic fitness, school commute mode and school commute distance. For study aim 2, multivariate logistic regression analyses assessed associations between parent perceptions of the neighborhood environment and ASC controlling for school commute distance and IMD. Statistical significance was set to *p* ≤ 0.05. All analyses were conducted using IBM SPSS Statistics version 23 (IBM, Armonk, NY, USA).

## 3. Results

Of the 217 children who returned written parental informed consent and participant assent, six participants were not present on the day of testing, and a further 17 children had incomplete data. Thus, data were available from 194 children (107 girls) (35.3% response rate). Participant characteristics are presented in Table 1. Preliminary analyses revealed no significant differences between included and excluded participants. Boys were taller (*p* < 0.05) and aerobically fitter than girls (*p* < 0.01) who were closer to maturation than boys (*p* < 0.001). More children commuted to school actively (52.6%) than passively (47.4%). Walking was the most common mode of commuting to school (47.4%), followed by car (44.8%), cycle (4.1%), bus (2.1%), scooter (1.0%), and other (0.5%). Active school commuters had significantly higher BMI (*p* = 0.02), BMI *z*-score (*p* = 0.05) and waist circumference (*p* = 0.01) than passive school commuters (Table 2). Differences were also observed for CRF but these did not reach statistical significance (*p* > 0.05). Children that lived closer to school had higher BMI, BMI *z*-scores and waist circumference but these did not reach statistical significance (*p* > 0.05).

Table 3 presents OR for deprivation, CRF, and weight status by school commute mode. Children who used passive transport were more likely to be classified as healthy weight (OR = 2.17, 95% CI = 1.10–4.30), aerobically fit (OR = 2.23, 95% CI = 1.20–4.14), and live further away from school (>0.5 km, OR = 38.14, 95% CI = 5.08–286.62; >1.0 km, OR = 11.61, 95% CI = 5.83–23.10), compared with children who commuted actively.

Table 4 presents OR for school commute mode, CRF, weight status and distance from school by deprivation group. Compared with children who lived in areas of HD, MD children were more likely to commute to school passively (OR = 2.41, 95% CI = 1.35–4.30), live further away from school (<0.5 km, OR = 2.95, 95% CI = 1.28–6.82; <1.0 km, OR = 2.06, 95% CI = 1.16–3.68), be classified as healthy weight (OR = 2.74, 95% CI = 1.37–5.48), and aerobically fit (OR = 2.52, 95% CI = 1.35–4.70).

ASC was positively associated with street connectivity (B = 0.62, OR = 1.66, 95% CI = 1.16–2.96) and land-use mix diversity (B = 0.55, OR = 1.86, 95% CI = 1.01–2.73), and was inversely associated with neighborhood aesthetics (B = −0.44, OR = 0.65, 95% CI = 0.44–0.95; Table 5).

## 4. Discussion

This study examined the association between active school commuting (ASC), body mass index (BMI) and cardiorespiratory fitness (CRF) in Liverpool schoolchildren. Counter to what might be assumed, we found that ASC was associated with higher BMI and lower CRF. The most recent systematic review in this area found that only 35.9% of included studies observed more favorable weight status among active school commuters relative to passive school commuters [14]. Fewer studies have reported an inverse relationship between ASC and child weight status [17,18]. There are several potential reasons for the inverse relationship found in this study.

Firstly, as observed here, children that commute to school actively tend to be from deprived backgrounds [17,44,45], and deprived children are more likely to live in an *obesogenic* environment that encourages the consumption of unhealthy food and/or discourages physical activity, placing them at greater risk of obesity compared to affluent children [46,47,48,49]. The indices of multiple deprivation (IMD) captures a range of deprivation markers including the neighborhood environment [35]. In Liverpool, high-deprivation (HD) neighborhoods could be considered *obesogenic*, as they are less walkable and have less access to self-contained gardens/yards compared to high-to-medium deprivation (MD) neighborhoods [34]. Moreover, HD children are more likely to experience an unbalanced diet at home [50], and be exposed to fast food and takeaway outlets along the home–school commute route [51,52], both of which are strong predictors of fatness [53,54]. To improve child health and foster more equitable neighborhoods requires an appreciation of the social determinants of health, and a structural approach to health promotion, through modifications to the physical, social, political, and economic environment in which children and families make health-related decisions [55,56]. Such changes may include but are not limited to improved infrastructure (e.g., sidewalks, bike lanes, and green spaces) and policy implementations (i.e., restrictions on fast food outlets and food marketing, and greater accessibility to affordable, healthy foods).

This study found an inverse association between ASC and CRF after deprivation was accounted for. Some previous studies have reported contrasting findings to those reported here [15,21,57]. However, these studies comprised a higher proportion of cyclists and observed higher CRF among cyclists compared to walkers and passive commuters [15,57]. In the present study, only 4.1% of children reported cycling to school. The average trip distance for cyclists is often greater than that of walkers and tends to be a more vigorous intensity activity [58]. It is well-established that high intensity PA (≥6 METs) is necessary to improve children’s CRF [59]. Walking is often performed at a moderate or light intensity, and thus, is unlikely to place the cardiorespiratory system under the necessary strain to confer positive adaptations to CRF. Presently, there is limited evidence for the association between walking to school and CRF among schoolchildren. Our findings add to the developing body of evidence.

Children that commuted actively to school lived closer to school than passive commuters. School to home commute distance is the strongest predictor of ASC [24,25]. D’Haese et al. [24] found that the criterion distance for walking to school in Belgium schoolchildren was 1.5 km. Chillón and colleagues [60] found that a distance of 1.4 km best discriminated walkers from passive commuters in a UK study involving 10-year-old schoolchildren. School choice can significantly reduce opportunities for ASC and thus impact on strategies to promote ASC. Schoolchildren live further from school than ever before. Presently, less than half of all English schoolchildren attend their nearest school [9]. Current educational policies in the UK are counterintuitive to public health goals of increasing child PA, especially ASC, for example, permitting schools to enroll schoolchildren from wide catchment areas thus creating long commuting distances. In such contexts, efforts to promote widespread adoption of ASC may be unrealistic. The uptake and maintenance of ASC is likely to be dependent on government policies aligning with public health priorities, as well as community and societal level influences to create safe and feasible commuting routes.

This study found that after adjusting for area deprivation and distance to school, neighborhoods perceived by parents as having well-connected streets, mixed land-use, and unpleasant aesthetic features were associated with a higher likelihood of ASC. In contrast to previous research [61,62], we observed an inverse association between neighborhood aesthetics and ASC. Our study is the first to investigate the association between ASC and parents’ perceptions of various neighborhood attributes in UK schoolchildren. Previous studies were undertaken outside of the UK, did not adjust for distance to school [61], and were based on ASC among adolescent girls [62]. It is plausible to suggest that favorable neighborhood aesthetics (e.g., well maintained sidewalks, green spaces, low volumes of street litter and graffiti) may improve children’s satisfaction of walking to school. However, many children in this study lived close to school and in neighborhoods classified as high deprivation. Whilst we cannot be certain that these children were from deprived backgrounds, deprivation is inversely associated with car access [63,64], and thus may result in these children having no other option but to commute to school actively.

In agreement with previous research [65], we found that neighborhoods perceived by parents as having a well-connected street network with numerous intersections/crossings were positively associated with ASC. These neighborhood features result in shorter and more direct commute routes to school, which is a well-established predictor of ASC [24,26]. Moreover, routes to school that are more direct and well-connected and made up of minor rather than major roads are likely to be perceived by parents as safer and thus more conducive to ASC given that they experience less motorized traffic and are subject to lower speed limits [29,66]. The introduction of traffic calming measures within school catchment areas such as pedestrianization and street crossings would provide a more conducive environment for children’s ASC and should be considered by future urban planners. Land-use mix diversity was also positively associated with ASC. A potential reason for this finding may be that neighborhoods with diverse land uses experience more people walking around the neighborhood and are thus more likely to be perceived by parents as safer [67]. Kerr et al. [61] and Larsen et al. [68] both found a positive relationship between land-use mix and ASC whereas Ewing et al. [69] reported contrasting findings. Further research is warranted to better understand how mixed land uses influences ASC.

Consistent with prior UK research, we found that children from highly deprived neighborhoods are most likely to commute to school actively [10,11,12]. One reason for this is that children from deprived neighborhoods are less likely to live in a family that owns a car [63,64]. Deprived children therefore commute to school actively in most part by necessity rather than choice. The distinction though between necessity and choice with regards to ASC is seldom explored in the literature. Of particular interest is the potential psychological strain placed on children and in the case of younger children, their parents, through relying on such forms of transport in often-unpleasant environments [70,71]. This could impact negatively on children’s motivation to participate in PA, especially walking for leisure in both the short and long-term. Further qualitative research is warranted to explore children’s perceptions of ASC, including the benefits and challenges they experience.

Importantly, it is not our intention to suggest that ASC is detrimental for Liverpool schoolchildren’s health. Rather, Liverpool schoolchildren with poorer health because they are deprived are more likely to commute actively to school, for reasons that warrant further investigation. Rather than advocating for those that participate [deprived children] to actively commute more to improve their weight status, we suggest that the challenge remains to identify ways to reduce deprivation, and increase ASC prevalence among the nonparticipants, especially those that live in close proximity to school. A recent UK study [72] exploring the habitual PA behaviors of a nuclear and single parent family, found that the nuclear family used the family car for short commute distances including the home to school commute (1.1 km). Future studies should consider recruiting such passive commuters that reside close to home to understand their decision to not commuting actively.

This is the first study to explore the influence of neighborhood attributes on schoolchildren’s ASC using the NEWS-Y survey. Several limitations are though, worthy of consideration. Our study used cross-sectional data, which limits inference of causality. When compared with the national average, children in this study lived in more deprived areas and had higher BMI. Therefore, generalizing our findings to more affluent and rural areas of the UK should be done with caution. The NEWS-Y survey is a valid and reliable measure of neighborhood attributes [32] but may be open to bias from respondents. The IMD is a well-established measure of deprivation reflecting a range of deprivation markers, but may not have accurately reflected the actual deprivation level of all participating schoolchildren. We did not assess sedentary time or energy intake, which both contribute to energy balance. Moreover, the relatively small sample size and low participant response rate may have biased results with active children more likely to have taken part in the study. Furthermore, we did not explore questions of context, which limits discussion on children’s reasons for commuting actively or passively to school. Although commute distance was measured objectively, this may not accurately reflect *actual* commute distance taken for all children. Another limitation is the fact that some children can be driven to school in the morning but commute actively in the afternoon. However, we did not distinguish between active, passive or ‘mixed transport’ commuters. Despite these limitations, the findings reported here are consistent with larger-scale studies [17,18].

## 5. Conclusions

In this study, schoolchildren who lived in more-deprived neighborhoods perceived by parents as being highly connected, unaesthetic and having mixed land-use were more likely to commute to school actively. These children were at greatest risk of being obese and aerobically unfit. Our findings suggest that deprivation may explain the counterintuitive relationship between obesity, cardiorespiratory fitness (CRF) and active school commuting (ASC) in Liverpool schoolchildren. These findings encourage researchers and policy makers to be mindful of the social determinants of health when planning and advocating behavioral and environmental health interventions. Further research exploring contextual factors to ASC, and examining the concurrent effects of ASC and diet on weight status by deprivation is needed. To improve child health and alleviate deprivation requires a systems approach to health promotion and actions on inequalities in wider social determinants operating outside the health system.

## Figures and Tables

**Table 1:** Participant characteristics (mean ± SD).

Variable	All (*n* = 194)	Boys (*n* = 87)	Girls (*n* = 107)
Age	9.96 (0.30)	9.97 (0.30)	9.95 (0.30)
Stature (cm)	139.12 (7.30)	140.42 (6.99)	138.06 (7.41) *
Mass (kg)	35.01 (8.44)	35.68 (7.68)	34.45 (9.01)
BMI (kg/m^2^)	17.92 (3.20)	17.96 (2.90)	17.89 (3.43)
Weight status (%)			
Normal weight	75.30	79.30	72.00
Overweight/obese	24.70	20.60	28.00
BMI *z*-score	0.32 (1.25)	0.51 (1.16)	0.16 (1.30)
Waist circumference	63.84 (7.72)	64.57 (7.97)	63.24 (7.50)
APHV	−2.64 (0.93)	−3.49 (0.45)	−1.94 (0.57) ***
CRF (shuttles)	38.18 (19.37)	48.37 (20.05)	29.90 (14.22) ***
Aerobically fit (%)	67.00	77.00	58.90 **
Commute distance (km)	1.68 (1.77)	1.60 (1.53)	1.74 (1.95)
School commute mode (%)			
Active	52.60	52.90	52.30
Passive	47.40	47.10	47.70
IMD score	36.80 (18.20)	36.87 (19.62)	36.73 (17.05)
NEWS-Y	0.03 (3.16)	0.05 (3.19)	0.02 (3.15)

APHV: age from peak height velocity; BMI: body mass index; CRF: cardiorespiratory fitness; NEWS-Y: neighborhood environment walkability scale-youth; IMD: indices of multiple deprivation. Significant gender difference at * *p* < 0.05; ** *p* < 0.01; *** *p* < 0.001.

**Table 2:** MANCOVA analyses of health-related variables by school commute mode group, adjusted for gender, APHV and school commute distance.

Variable	Active Mean (95% CI) (*n* = 102)	Passive Mean (95% CI) (*n* = 92)	*p* Value
BMI	18.33 (17.79–18.87)	17.32 (16.75–17.89)	0.02
BMI *z*-score	0.45 (0.23–0.67)	0.12 (−0.11–0.36)	0.05
Waist circumference	64.84 (63.57–66.11)	62.29 (60.95–63.64)	0.01
CRF	37.98 (34.37–41.60)	38.99 (35.16–42.84)	0.72

MANCOVA: multivariate analysis of covariance; BMI: body mass index; CI: confidence interval; CRF: cardiorespiratory fitness.

**Table 3:** OR (95% CI) for likelihood of being classified as healthy weight, aerobically fit, and living within 1 km from school by school commute mode.

Variable	Active Mean (95% CI) (*n* = 102)	Passive Mean (95% CI) (*n* = 92)	*p* Value
Healthy weight	47.9%	52.1%	0.02
	2.17 (1.10–4.30)		
Aerobically fit	46.2%	53.8%	0.01
	2.23 (1.20–4.14)		
Commute distance			
<0.5 km	30.0%	1.1%	<0.001
	38.14 (5.08–286.62)		
<1.0 km	73%	18.9%	<0.001
	11.61 (5.83–23.10)		

OR: Odds ratio.

**Table 4:** OR (95% CI) for likelihood of being classified as healthy weight, aerobically fit, an active commuter and living with 1 km from school by deprivation group.

Variable	MD Mean (95% CI) or % (*n* = 96)	HD Mean (95% CI) or % (*n* = 98)	*p* Value
Healthy weight	84.4%	66.3%	<0.01
	2.74 (1.37–5.48)		
Aerobically fit	77.1%	57.1%	<0.01
	2.52 (1.35–4.70)		
Commute distance			
<0.5 km	9.4%	23.4%	0.01
	2.95 (1.28–6.82)		
<1.0 km	38.5%	56.4%	0.01
	2.06 (1.16–3.68)		
Active commute	36.7%	63.3%	<0.01
	2.41 (1.35–4.30)		

**Table 5:** Associations between neighborhood environment attributes and ASC.

Variable	B	SE	OR (95% CI)	*p* Value
Land-use mix diversity	0.62	0.24	1.86 (1.16–2.96)	0.01
Constant	−1.80	0.73	0.17	0.01
Street connectivity	0.50	0.26	1.66 (1.01–2.73)	0.04
Constant	−1.45	0.76	0.23	0.06
Neighborhood aesthetics	−0.44	0.19	0.65 (0.44–0.95)	0.02
Constant	1.13	0.51	3.09	0.03

B: unstandardized β coefficient; SE: standard error; OR: odds ratio; OR = exp (β). Adjusted for IMD and school commute distance. Only variables that showed a statistically significant association with ASC are presented.
